# Crystal Structure of an Active Form of Human MMP-1

**DOI:** 10.1016/j.jmb.2006.06.079

**Published:** 2006-09-08

**Authors:** Shalini Iyer, Robert Visse, Hideaki Nagase, K. Ravi Acharya

**Affiliations:** 1 Department of Biology and Biochemistry, University of Bath, Claverton Down, Bath BA2 7AY, UK; 2 Department of Matrix Biology, Kennedy Institute of Rheumatology Division, Faculty of Medicine, Imperial College London, 1 Aspenlea Road, London W6 8LH, UK

**Keywords:** MMP, matrix metalloproteinase, ECM, extracellular matrix, matrix metalloproteinases, fibroblast collagenase, collagen, X-ray crystallography, inhibitor-free

## Abstract

The extracellular matrix is a dynamic environment that constantly undergoes remodelling and degradation during vital physiological processes such as angiogenesis, wound healing, and development. Unbalanced extracellular matrix breakdown is associated with many diseases such as arthritis, cancer and fibrosis. Interstitial collagen is degraded by matrix metalloproteinases with collagenolytic activity by MMP-1, MMP-8 and MMP-13, collectively known as the collagenases. Matrix metalloproteinase 1 (MMP-1) plays a pivotal role in degradation of interstitial collagen types I, II, and III. Here, we report the crystal structure of the active form of human MMP-1 at 2.67 Å resolution. This is the first MMP-1 structure that is free of inhibitor and a water molecule essential for peptide hydrolysis is observed coordinated with the active site zinc. Comparing this structure with the human proMMP-1 shows significant structural differences, mainly in the relative orientation of the hemopexin domain, between the pro form and active form of the human enzyme.

## Introduction

Connective tissue remodeling is a complex process involving a plethora of cytokines, growth factors, and turnover of extracellular matrix (ECM). The main enzymes that degrade ECM molecules are matrix metalloproteinases (MMPs), which are also known as matrixins. Under normal physiological conditions, MMP activity can be regulated at various stages: during transcription, proteolytic processing of their inactive pro forms, zymogens, as well as by inhibition of enzyme activity by endogenous inhibitors such as tissue inhibitors of metalloproteinases or TIMPs.^1^^,^^2^ These enzymes have a similar domain structure: an N-terminal signal sequence to target for secretion, a pro-peptide domain to maintain latency, a catalytic domain containing the catalytic zinc, a linker region, and a C-terminal four-bladed propeller structure called the hemopexin domain. Some of the MMPs have additional domains, e.g. the fibronectin repeats in gelatinases. These domains are important in substrate recognition and in inhibitor binding.^3^ The human MMP family to date comprises of about 23 enzymes that are classified based on their preferred substrate and cellular localisation: collagenases, gelatinases, stromelysins, elastase, membrane-type MMPs and so forth.^4^

Collagenases (MMP-1, MMP-8 and MMP-13) are the key enzymes that are capable of cleaving interstitial fibrillar collagen. Apart from these enzymes MMP-2 (gelatinase A) and MMP-14 (MT1-MMP) are also able to initiate the breakdown of collagen fibrils.^5^^,^^6^ Interstitial collagens I, II and III are triple-helical proteins that are the essential structural components of all connective tissues such as the cartilage, bone, skin, tendons and ligaments. These extracellular glycoproteins provide scaffolding of the tissue and play an important role in cellular processes such as cell migration, proliferation and differentiation. Physiological collagenolysis is integral to several biological processes such as embryogenesis, tissue repair and remodeling, angiogenesis, organ morphogenesis and wound healing.7., 8., 9. The collagenases cleave the triple-helical collagen approximately three-quarters away from the N terminus of the substrate, resulting in three-quarters and one quarter length fragments that are unstable at body temperature and undergo denaturation, rendering them susceptible to other non-specific tissue proteinases. However, under aberrant circumstances degradation of collagen results in pathological conditions such as cancer, atherosclerosis, arthritis, aneurysm and fibrosis.^8^^,^^10^

The proteolytic activity of the enzyme resides in the catalytic domain but it requires the hemopexin domain in order to cleave the three chains of the triple-helical collagen.^11^ The crystal structure of porcine MMP-1,^12^ determined sometime ago, revealed details of the active site structure and the specificity pocket but this structure does not shed any light on how collagenolytic MMPs can cleave the triple-helical collagen. Recently, the X-ray structure of human proMMP-1 (MMP-1 zymogen) was elucidated.^13^ The structure revealed the interaction between the pro-peptide and the hemopexin domain of the enzyme, which results in a “closed” conformation of the zymogen in contrast to the “open” conformation of catalytic domain of the active MMP-1.

The three-dimensional structure of the human MMP-1 (E200A), an active site mutant, reported here takes us a step closer towards a more complete understanding of the interaction of the collagenases with the triple-helical collagens. It reveals new features of the active protease and provides a platform for understanding the structural changes that accompany zymogen activation.

## Results

### Overall structure

**Figure 1:**
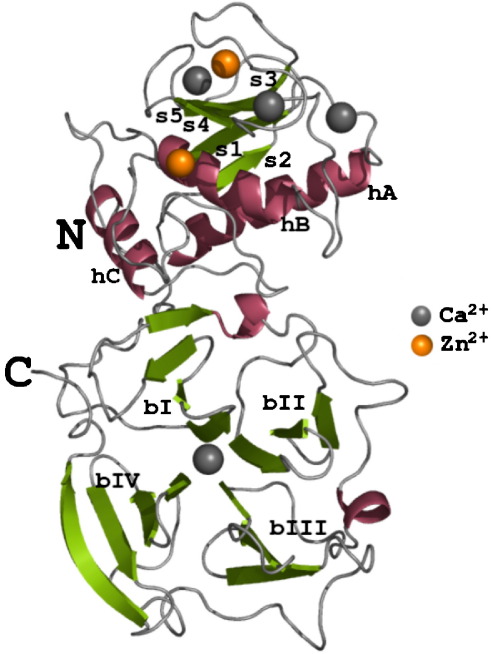
Ribbon representation of the three-dimensional structure of human MMP-1 (E200A). Helices have been coloured pink and the strands shown in green. There are four calcium ions and two zinc ions found in the structure that have been coloured grey and orange, respectively. The secondary structural elements have been annotated: helices (hA-hC), strands (s1–s5) of the catalytic domain and blades (bI–bIV) of the hemopexin domain.

The structure of human MMP-1 (E200A) was determined at 2.67 Å resolution with two monomers (chains A and B) in the asymmetric unit of the trigonal space group, *P*3_2_21 (see Table 1 for crystallographic statistics). The overall domain structure of human MMP-1 is similar to that of the previously solved full-length enzymes (Figure 1). The structure comprises of the N-terminal catalytic domain, the linker region and the C-terminal hemopexin domain. The catalytic domain of one monomer contacts the hemopexin domain of the other monomer. Interestingly, the contact site used by the two monomers in the asymmetric unit to form the dimer is not the same as the dimerisation site observed in the proMMP-1 structure^13^ or that for the MMP-9 hemopexin domain.^14^ This indicates that the dimerisation mechanism is perhaps not a general rule of thumb for the hemopexin domains and is most probably a crystallisation artefact. Monomers A and B deviate from each other with an overall r.m.s. deviation of 0.87 Å (for 367 C^α^ atoms). The dimer is stabilised by eight hydrogen bonds and 54 van der Waals contacts (Table 2). These contacts are facilitated by a total of 33 residues (18 residues from monomer A and 15 residues from monomer B). Only nine amino acid residues are common to both the monomers: Pro104, Asp105, Leu106, Arg183, Trp184, Thr185, Val300, Phe301 and Gln335. Apart from the asymmetry seen in the residues that participate from each monomer at the dimer interface, we also observe a non-symmetric interaction pattern. However, it is believed that this dimer is not physiologically relevant, as we found that human MMP-1 is a monomer in solution (R.V. & H. N., unpublished results).

**Table 1:** Crystallographic data processing and refinement statistics

Space group	Trigonal, *P*3_2_21
Unit cell dimensions (Å)	*a* = *b* = 138.48, *c* = 110.05
Resolution range (Å)	24.67–2.67
Total reflections measured	227,517
Unique reflections measured	30,817
*R*_*sym*_ (%)^a^	8.0 (35.8)
*I*/σ(*I*) (outermost shell)^b^	22.6 (4.7)
Completeness (outermost shell) (%)	91.8 (91.2)
*R*_*cryst*_ (%)^c^	22.3
*R*_*free*_ (%)^d^	25.9

A. *Contents of the asymmetric unit*	
Protein atoms	5798
Solvent molecules	198
Ions	12 (4 zinc and 8 calcium)

B. *r.m.s. deviation*	
Bond lengths (Å)	0.007
Bond angles (°)	1.35

C. *Average* B*-factor (Å*^*2*^*)*	
All atoms (monomers A and B)	44.7 (A); 49.9 (B)
Main-chain atoms	44.4 (A); 50.0 (B)
Side-chain atoms	45.0 (A); 49.8 (B)
Ions (zinc and calcium)	44.9
Solvent molecules	34.1
Overall *B*-factor (Å^2^/Da) (from Wilson plot)	63.1

a *R*_sym_ = ∑_*hkl*_∑_*i*_|*I*_*i*_(hkl−<I(*hkl*)>|/∑_*hkl*_∑_*i*_*I*_*i*_*(hkl),* where *<I>* is the averaged intensity of the *i* observations of reflection *hkl*.

b Outermost shell: the resolution range of the outermost shell is 2.77–2.67 Å.

c *R*_cryst_ = ∑||*F*_o_|*−*|*F*_c_||/∑|*F*_o_|, where *F*_o_ and *F*_c_ are observed and calculated structure factors, respectively.

d *R*_free_ is equal to *R*_cryst_ for a random subset of reflections (2.2%) not used in refinement.^54^

**Table 2:** van der Waals contacts at the dimer interface

Monomer A	Monomer B	No of contacts
Pro104	Gln333, Gly334^(2)^	3
Asp105	Gln333, Gly334^(2)^, Gln335^(4)^, Asn336^(3)^	10
Leu106	Gln333	2
Pro158	Val300^(2)^, Phe301^(2)^	4
Glu180	Gln335	1
Asp181	Phe301, Gln335	2
Glu182	Gln335	4
Arg183	Pro303^(2)^, Gln335^(10)^, Asn336	13
Thr185	Asn336	1
Arg189	Gln304	5
Arg272	Pro104, Leu106^(2)^, Pro107	4
Glu274	Pro104	1
Arg285	Pro104^(2)^, Asp105	3
Val300	Arg183	2
Phe301	Asp105^(4)^, Arg183	5
Gln335	Asp105^(2)^, Leu106, Arg183^(2)^, Thr185	6

Potential hydrogen bonds between monomers A and B at the dimer interface					
Monomer A	B-factor (Å^2^)	Monomer B	B-factor (Å^2^)	Distance D… A (Å)	Angle D–H… A (°)
Asp105 O	25.3	Asn336 Nδ2	23.2	2.9	144.2
Trp184 O	14.9	Asn336 Nδ2	22.1	2.9	167.2
Arg272 Nη1	34.9	Pro104 O	39.8	3.1	151.0
Arg285 Nη1	18.2	Asp105 Oδ1	18.4	3.3	166.3
Glu294 ε1	21.3	Arg146 Nη1	15.2	3.1	124.1
Val300 O	17.5	Arg183 Nη1	20.4	2.9	169.4
Gln335 Nε2	32.3	Asp105 O	18.8	3.3	168.9
Gln335 Nε2	22.6	Trp184 O	22.3	2.8	159.8

Hydrogen bond interactions were identified with the program HBPLUS^31^ The upper limit for the donor–acceptor distance was 3.3 Å, except for contacts involving a sulphur atom (limit 3.6 Å); the lower limit for the donor–hydrogen–acceptor angle is 120°. Bond angles are not given where the hydrogen position is ambiguous. Contact distances are the maximum allowed values of C–C, 4.1 Å; C–N, 3.8 Å; C–O, 3.7 Å; O–O, 3.3 Å; O–N, 3.4 Å; N–N, 3.4 Å; C–S, 4.1 Å; O–S, 3.7 Å; N–S, 3.8 Å. The contact distances were calculated using CONTACT.^26^

### Catalytic domain

**Figure 2:**
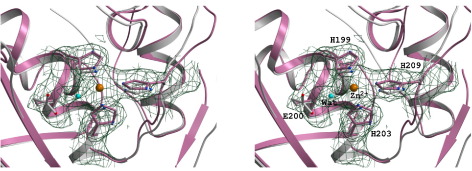
Structure of the active site of the E200A variant of human MMP-1 superimposed with the wild-type enzyme (PDB code: 1CGL).^20^ The mutant enzyme is in pink and the wild-type enzyme in grey. The water molecule at the catalytic site that is important for peptide bond hydrolysis is shown in cyan and the catalytic zinc is displayed in orange. Electron density (2*F*_o_-*F*_c_ map contoured at 1.0σ) is shown around the catalytic site residues, the catalytic zinc and the water molecule.

The structure of MMP-1 catalytic domain is very similar to those of other MMPs. The metalloproteinase domain is about 160 amino acid residues in length with the catalytic zinc ion residing in the C-terminal segment of this domain. The catalytic fragment of the protease consists of three α-helices and a highly twisted five-stranded β-sheet. The active site zinc is bound in the sequence HELGHXXGXXH by the three His residues: His199, His203, His209 and a water molecule at the active site cleft (Figure 2). This is the first MMP-1 structure where a water molecule essential for peptide hydrolysis is observed at the active site because all the previous structures of the metalloproteinase domain of MMP-1 are in the inhibited state of the enzyme.

Also observed in this domain is the salt bridge between the ammonium group of the N-terminal, Phe81 and the carboxylate group of the side-chain of Asp232. The generation of the correct N terminus (Phe81) in the activation process of proMMP-1 is crucial for the enzyme to have full activity against collagen.^15^ If the N terminus is either longer or shorter the activity against collagen drops to 30–40%.^15^ Only with the correct N terminus the formation of the salt bridge is possible, and this stabilises the structure of the N terminus as was originally shown for MMP-8.^16^^,^^17^

**Figure 3:**
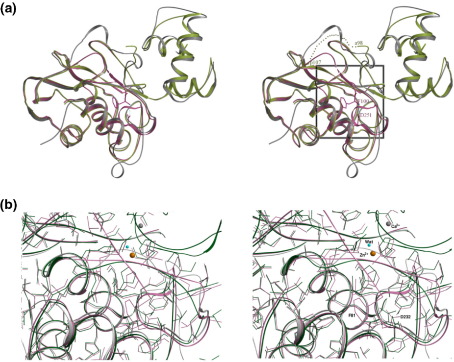
Stereo view of the comparison of procollagenase-1 with the active enzyme. (a) Active human MMP-1 (pink), procollagenase-1 (green; PDB code: 1SU3) and prostromelysin-1 (grey; PDB code: 1SLM) have been superimposed to highlight the major movement of Phe81 (N-terminal residue of active enzyme; Phe83 in active stromelysin-1) upon activation. Also shown is the resulting salt-bridge between Phe81 and Asp232. The dotted green line represents the likely trajectory the N-terminal segment (81–87) would take in the procollagenase-1. The prostromelysin-1 structure was superimposed to help visualise this movement as this segment in the procollagenase-1 structure is disordered. (b) Close-up of the N-terminal area (the area boxed in (a). Colour coding for the structures is the same as that followed for (a).

Alignment of the pro-enzyme (1SU3) structure with that of the mature enzyme (2CLT here) shows Phe81 to have moved some 17 Å from its original position in the zymogen form of collagenase-1 (Figure 3(a)). The structure of proMMP-3 (PDB code: 1SLM)^18^ was aligned as well to help calculate the displacement of Phe81. The first seven N-terminal residues were not observed in the proMMP-1 structure because of lack of proper visible electron density.^13^ This deviation gradually decreases and by residue Pro88 the two structures are in register and remain remarkably similar throughout the catalytic domain. There are only slight differences, if any, in the side-chain orientation of the N-terminal residues in the catalytic domain cleft (Figure 3(b)).

The structural zinc exhibits tetrahedral coordination facilitated by residues His149, Asp151, His164 and His177. The catalytic domain of MMP-1 also contains three calcium-binding sites. Either four or five liganding residues coordinate all the calcium ions, except one in the catalytic domain of monomer A (Table 3). One calcium ion packs the S-loop (between strands s3 and s4) against the liganding residues from strand s5. The second calcium ion of the catalytic domain is sandwiched between strand s3 and the loop containing strands s4 and s5. The third calcium site is determined by the presence of the critical residue, Asp105 that comes from the loop following strand s1. The other residues (Glu180 and Glu182) that provide coordination to this calcium ion come from the loop following strand s5. One interesting feature observed in this crystal structure by virtue of dimerisation is that residue Gln335 from monomer B contributes the fourth coordinating ligand for this calcium ion. This feature, however, is not replicated in the corresponding calcium-binding site in monomer B. Details of the metal site geometries can be found in Table 3.

**Table 3:** Metal site geometries (distance in Å)

Metal site	Coordinating residue	Monomer A	Monomer B
Catalytic Zinc	His199 NE2	2.19	2.19
	His203 NE2	2.13	2.23
	His209 NE2	2.20	1.99
	Water	2.32	2.31
Structural Zinc	His149 NE2	2.15	2.32
	Asp151 OD2	2.08	2.22
	His164 NE2	2.21	2.17
	His177 ND1	2.09	2.12
Calcium 1	Asp105 OD2	2.40	2.43
	Glu180 O	2.27	2.35
	Glu182 O	2.40	2.39
	Gln335 OE1 (B)	2.28	–
	Glu180 OE2	–	2.14
Calcium 2	Asp139 O	2.42	2.29
	Gly171 O	2.29	2.25
	Gly173 O	2.05	2.11
	Asp175 OD2	2.30	–
	Water	2.42	2.30
Calcium 3	Asp156OD2	2.26	2.44
	Gly157 O	2.42	–
	Gly159 O	2.03	2.29
	Asn161 O	–	2.40
	Asp179 OD2	2.39	2.35
	Glu182 OE2	2.43	2.23
Calcium 4	Asp266 O	2.36	2.31
	Glu310 O	2.34	2.41
	Asp359 O	2.48	2.37
	Asp408 O	2.67	2.45

### Linker region

**Figure 4:**
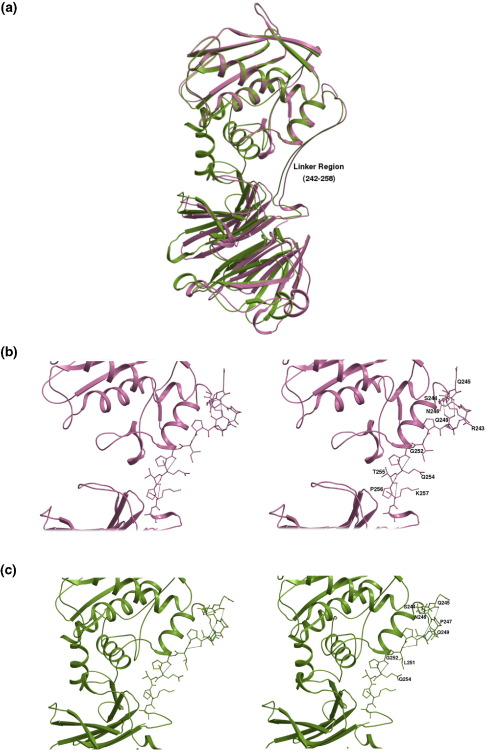
Comparison of the hydrogen-bonding interactions within the linker region. (a) Superposition of active MMP-1 (pink) and procollagenase-1 (green) to highlight the conformational similarity of the linker region in the two structures. (b) Stereo view of the linker region in active MMP-1 showing the hydrogen-bonding interactions between the residues. (c) Stereo view of the linker region in procollagenase-1 showing the hydrogen bonds within the region.

In MMPs the catalytic domain is followed by a stretch of 15–65 amino acid residues referred to as the linker or the hinge region. This region is rich in proline residues and replacement of those with alanine drastically reduced the collagenolytic activity of MMP-8 (neutrophil collagenase),^19^ indicating that the presence of the correct linker structure is important for collagenolysis. This region in MMP-1 with 16 residues is well defined in the present structure unlike that in the full-length crystal structure of porcine collagenase. The conformation of the linker region is quite similar in both proMMP-1^13^ and the present MMP-1 structures (Figure 4(a)). The residues of the linker region make extensive contacts (six hydrogen bonds and 73 van der Waals contacts; Table 4) with both the catalytic and the hemopexin domain of the enzyme. These interactions stabilise the domain arrangement in MMP-1, which is required for the concerted action of the two domains. Comparing the interactions of the linker region with the catalytic and the hemopexin domain in both the pro-enzyme and the active enzyme did not reveal many differences. Most of the interactions seen in proMMP-1 (seven hydrogen bonds and 71 van der Waals contacts) are still observed in the present structure, indicating that the activation of the enzyme does not result in structural changes and the overall structure of the areas around the linker region are mostly conserved. Interestingly though, the hydrogen-bonding interactions (Table 4) within this region are quite different between the two forms of the molecule. There are a total of seven hydrogen bonds within the linker region of active MMP-1 (Figure 4(b)) compared to the five observed in proMMP-1 (Figure 4(c)), with only two bonds common to both structures This difference in the number of hydrogen bonds is perhaps not of much significance when seen in the light of the extensive contacts this region makes with the other two domains.

**Table 4:** Linker region interactions in human MMP-1 and proMMP-1

van der Waals interactions		
Residue	MMP-1 interactions	proMMP-1 interactions
Gly242	Pro127, Gln238^(8)^, Tyr241	Thr126^(2)^, Pro127^(2)^, Gln238^(7)^, Ile240, Tyr241
Arg243	Pro127, Gln238^(6)^	Thr126, Pro127, Gln238^(2)^
Ser244	Asn124, Val125^(5)^	Asn124, Val125^(2)^, Thr126^(3)^
Gln245	Pro127	–
Pro250	Asn124	Asn124, Val125^(2)^
Ile251	Gln231, Ile234^(2)^	Ile234
Gly252	Leu229, Gln231^(7)^, Ile234^(2)^	Leu229, Gln231, Ile234^(2)^
Pro253	Leu229, Ala230, Arg281^(2)^	Gln228^(5)^, Leu229, Ala230^(2)^, Arg281^(4)^
Gln254	Gln231^(2)^	Gln231^(2)^
Thr255	Asp212^(2)^, Ile213, Gly214, Leu295	Asp212^(4)^, Gly214, Phe282, Leu295
Pro256	Leu263, Lys279, Phe282^(5)^, Met284^(2)^	Phe282^(3)^, Met284
Lys257	Cys259	Cys259, Asp260^(3)^
Ala258	Lys257, Cys259^(3)^, Asp260, Asn442, Phe445^(6)^, Cys447^(2)^	Cys259^(3)^, Asp260, Leu263, Val293^2)^, Asn442^(2)^, Phe445^(2)^, Cys447

Hydrogen bonding interactions with the linker region		

Source	Target	Distance (Å)

Gly242 N	Gln238 O	2.66
**Gln249 OE1**	**Gln238 NE2**	**2.96**
Gly252 N	Gln231 OE1	3.09
Pro253 O	Gln231 N	2.98
Thr255 OG1	Asp212 OD1	2.64
***Lys257 O***	***Asp60 N***	***3.14***
***Lys257 O***	***Cys259 N***	***3.18***
Ala258 O	Asn442 ND2	3.02

Hydrogen bonding interactions within the linker region		
Source	Target	

Arg243 O	Glu245 N	
Ser244 N	Gln249 OE1	
Ser244 O	Gln249 NE2	
**Ser244 OG**	**Asn246 N**	
***Glu245 O***	***Pro247 N***	
***Asn246 O***	***Glu249 N***	
***Glu249 O***	***Pro251 N***	
**Gly252 O**	Glu254 N	
Gln254 O	Pro256 N	
Thr255 O	Lys257 N	

Interactions in bold font are common to both the active MMP-1 and the pro-enzyme; interactions in bold and italics are unique to the pro-enzyme; the rest are unique to the active MMP-1.

### Hemopexin-like domain

The hemopexin domain starts with Cys259 and forms a complete circle by joining to Cys447 in a disulphide bond that connects blade bI with blade bIV, giving this domain the characteristic four-bladed β-propeller structure. Each blade starts near the periphery with either the motif DAA or DAX, in which the Asp residues (Asp266, Asp359 and Asp408) coordinate the central calcium ion through their carbonyl oxygen atom. Glu310 provides the fourth coordination thus completing the acidic patch at the entrance of the central, solvent-accessible channel (Table 3). The side-chains of these residues form salt bridges to the neighbouring β-strands holding the entrance of the central channel together. Three water molecules are found trapped in the centre of this channel. These, however, are not involved in the coordination geometry of the calcium ion at the tunnel centre. Two of the water molecules are at positions corresponding to the sodium and chloride ion in the proMMP-1 structure.^12^ The water molecule corresponding to the sodium ion is at hydrogen-bonding distances to the carbonyl oxygen atom of Ile268, Ala312, Ala361 and Val410. The same cannot be said for the one corresponding to the chloride ion, which does not make any hydrogen bonds with the main-chain amides of the residues mentioned above. It is very likely that the presence of these ions is a consequence of the crystallisation conditions rather than a stability requirement for the hemopexin-like domain.

### Comparison with the porcine MMP-1 and the human proMMP-1 structures

**Figure 5:**
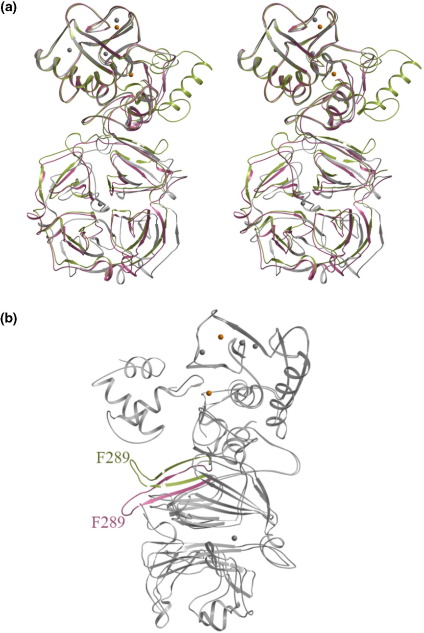
Conformational differences in the hemopexin-like domain of proMMP-1 and active MMP-1. (a) Shown in pink is human active MMP-1, in grey is porcine MMP-1 and green is for human proMMP-1. Superposition was based on the catalytic domain of all the three molecules. The stereo view highlights the relative movement of the hemopexin-like domain of proMMP-1 and active MMP-1. Activation of the pro-enzyme (green: closed configuration) results in an open-conformation (pink/grey: active MMP-1). (b) Major displacement of the Phe289-Tyr290-Pro291 loop of the hemopexin-like domain in the pro and active form of the human enzyme. Both forms of the enzyme have been coloured grey except for the displaced loop, which is shown in pink in the active enzyme and in green in the pro-enzyme. All the Pigures were made using the program MOLSCRIPT.^30^

Full-length human MMP-1, human proMMP-1^13^ and porcine MMP-1^12^ were superimposed on the basis of the catalytic domain of the three structures. Alignments were also done for just the hemopexin domain of the molecules. As expected both types of alignments gave the same outcome whereby the active form of the enzyme (human and porcine) were closer in their structure as opposed to the proMMP-1 structure. The average r.m.s. deviation over 367 C^α^ atoms of the porcine and human MMP-1 is ∼1.4 Å, whereas the r.m.s. deviation when aligning the full-length active MMP-1 with the proMMP-1 structure is 1.6 Å. If, however, we take only the hemopexin-like domain into consideration, then the conformational differences that lie within this domain are brought to light (Figure 5(a)). The superimposed porcine and human MMP-1 hemopexin-like domains vary by 1.2 Å (maximum displacement of 5.2 Å). This value increases to 1.7 Å when superimposing the hemopexin-like domains of the active human MMP-1 and the human proMMP-1. A considerable change in conformation is reflected in the residues that interact with the pro-domain. Most of these differences are by virtue of the Phe289-Tyr290-Pro291 region of the hemopexin-like domain. Table 5 shows these residues, their interactions and the shift in Cα position observed when the pro and active forms of human MMP-1 were superimposed. The largest movement of 16 Å is observed for the residue, Phe289 (Figure 5(b)). Such a perturbation in structural conformation is not evident in the catalytic domain of the enzyme (Table 5). It seems like Arg281 acts as a pivot around which the hemopexin-like domain undergoes displacement upon activation as suggested by Jozic *et al.*^13^

**Table 5:** Effects of interaction with pro domain residues

	Pro domain residues		Shift in C^α^ position (Å)
MMP-1 residues	van der Waals interactions	Hydrogen bonds	
Gly160	Pro71^(2)^, Arg72	Gly160 O–Arg72 N (2.9 Å)	0.87
Asn161	Arg72^(2)^, Gly74^(4)^	Asn161 ND2–Gly74 N (3.0 Å)	0.33
Leu162	Pro71^(2)^	Leu162 N–Arg72 O (2.9 Å)	0.33
Asn163	Cys73^(2)^, Gly74	Asn163 O–Gly74 N (2.8 Å)	0.09
His164	Val75	–	0.00
Ala165	Val75^(2)^	–	0.17
Tyr191	Pro71^(6)^	–	0.00
His199	Cys73^(4)^	–	0.36
His203	Cys73^(2)^, Val75^(3)^	–	0.50
His209	Phe51, Cys73^(4)^, Val75^(4)^, Pro76, Asp77	His209 ND1–Asp77 OD1 (2.7 Å)	0.44
Ser210	Phe51^(4)^	–	0.50
Thr211	Phe51^(11)^	–	0.79
Tyr218	Phe52^(4)^	–	0.85
Pro219	Phe51^(3)^, Phe52^(2)^, Arg72^(6)^, Asp77^(3)^	Pro219 O–Cys73 N (2.9 Å)	1.08
Ser220	Gln70^(2)^, Pro71, Arg72^(2)^,	–	1.18
Tyr221	Pro71	Tyr221 N–Pro71 O (3.0 Å)	1.27
Phe289	Lys55, Asp61, Glu63^(4)^	–	15.52
Tyr290	Gly53^(2)^, Leu54^(2)^, Glu63^(5)^	Tyr290 OH–Glu63 OE1 (2.6 Å)	11.88
Pro291	Gly53, Leu54, Lys55^(4)^	–	8.87

One region in the catalytic domain that generates interest is the region between Tyr218 and Tyr221. The shift in the Cα position between the active and the proMMP-1 is not as dramatic as that observed for the Phe289-Tyr290-Pro291 region but they are involved in several interactions with the pro domain around the cysteine-switch region. As a result when the pro-enzyme undergoes activation, this region of the catalytic domain opens wider and further exposes the crucial *cis*-configured Glu190-Tyr191 peptide in MMP-1.

## Discussion

### Reconciliation of structural findings: pro-collagenase *versus* active collagenase

MMPs are multi-domain enzymes that consist of a pro domain, catalytic domain, a linker region and a hemopexin-like domain. Cleavage of the pro domain leads to a substantial rearrangement of the N-terminal residues 81–88 (Figure 3(a)). The activation process swings Phe81 towards the proteinase domain and terminates with a salt link between the amine of Phe81 and the carboxylate side-chain of Asp232. This interaction imparts several-fold greater enzymatic activity to MMP-1 than those with either an extended or a shorter N terminus, which lack the salt linkage.^17^

Previously an inhibitor-free structure of the catalytic domain of MMP-1 was reported.^20^ This structure was, however, unique in the sense that the N-terminal Leu-Thr-Glu-Gly (83–86) residues of one molecule occupied the active site of the other molecule thus forming an unnatural inhibited complex, which should not really be considered as an inhibitor-free structure. The present structure on the other hand stands unique in being elucidated in the absence of an inhibitor. This is the first unliganded structure of the full-length MMP-1 where a water molecule is seen at the active site providing the fourth ligand for the catalytic zinc in the tetrahedral coordination sphere (Figure 2). It must be mentioned here that the present structure is an active site mutant where the catalytic glutamate residue has been mutated to an alanine. It is likely that the scissile peptide bond and the catalytic glutamate (Glu200) flank the active site water molecule on either side. The favourable hydrogen bond between Glu200 and the water molecule would make it more nucleophilic. The strongly polarised carbonyl group of the scissile bond (by virtue of its interaction with the catalytic zinc) would face a properly oriented water molecule. An unstable transition state results in the transfer of one water proton to the leaving nitrogen (the amino group of the scissile bond) *via* the Glu200 carboxylate shuttle. This is followed by the cleavage of the peptide bond and concomitant shuttle of another water proton to the amino group. This hypothesis will, however, need to be substantiated with a transition state structure.

The linker region is considered to play an important role in collagenolysis,^19^^,^^21^ but its exact role is not clear. The structures of both proMMP-1 and active MMP-1 have revealed the residues of this region to be in close contact with the catalytic and the hemopexin-like domain. Comparative analysis of the extensively similar contacts made by the residues of the hinge region reveal that despite being highly exposed with no secondary structure, the conformation of the linker peptide is perhaps a signature of its sequence and does not depend on the crystal packing (Figure 4(a)). Mutagenesis studies of this region result in decreased collagenolytic activity of MMP-1^21^ and MMP-8.^19^ This may be due to structural changes in this region affecting the interactions with the catalytic and hemopexin domains that are required for collagenolysis.^22^

The cleavage of the pro domain to form the active enzyme is accompanied by major conformational rearrangement in the residues that interact with the pro domain. Residues from both the catalytic domain and the hemopexin-like domain make interactions with the pro domain residues. The effects on the overall backbone structure of the catalytic domain is minimal; the effects on the hemopexin-like domain is, however, quite dramatic (Figure 5(a)). The Phe289-Tyr290-Pro291 region of the hemopexin-like domain undergoes the most significant conformational change (Figure 5(b)). In essence, the hemopexin-like domain undergoes a major displacement towards the catalytic domain, thus widening the cleft between the proteinase domain and the hemopexin-like domain on the active site face of the enzyme. This altered configuration makes the active site residues and the RWTNNFREY (residues 183–191) interface, a segment critical for collagenolysis,^23^ more accessible for native collagen.

### Implications for collagenolysis

Despite growing awareness of the importance of collagen-recognising determinants it is still unclear as to which part of the collagenase molecule makes the first contact with the triple-helical collagen. Several hypotheses have been suggested to try and explain the steps involved in collagenolysis (the most recent by Jozic *et al.*^13^). Docking studies were performed using a single-stranded, collagen α1(I)-like 15mer peptide with the Gly-Ile cleavage site incorporated in an attempt to better understand the functioning of the active enzyme. The peptide was modelled on the known structure of one of the chains of the triple-helical collagen (PDB code: 1BKV).^24^ Several solutions were generated. We used localised interactions as our guide to help us pick one possible solution: (a) contact of the carbonyl group of the Gly-Ile bond of the peptide with the active site determinants, especially the catalytic zinc; and (b) contact with the critical *cis*-configured Glu190-Tyr191 peptide. This solution revealed the peptide to be aligned to the continuous bulge-edge strand (Gly160-Phe166) and the wall-forming segment (Pro219-Phe223) in an extended manner. This solution, however, upon energy minimisation moved outside of the coordination sphere of the catalytic zinc but it was still close enough to be polarised by the catalytic zinc. Since the peptide was only 15 amino acid residues long, we could not see any interactions with the C-terminal hemopexin-like domain. It has been proposed that the triple-helical collagen would run *via* the Glu190-Tyr191 *cis*-peptide exosite making extensive interactions with the residues of the hemopexin-like domain.^22^

The structure of the active form of human MMP-1 has provided us insights on the conformational changes that occur upon activation of the pro-enzyme and these are valuable clues towards understanding the mechanism of collagenolysis. A better understanding of the mechanism of collagen cleavage will assist towards design of inhibitors that would specifically interfere with collagenolysis without affecting (beneficial) the cleavage of other substrates.

## Experimental Procedures

### Cloning, protein expression and protein purification

The catalytically inactive mutant proMMP-1 (E200A) was cloned, overproduced, refolded, and purified as described.^22^ A catalytic site mutant of this enzyme was chosen in order to prevent autocatalytic cleavage during crystallisation. The mutant zymogen was activated as described^22^ at a final concentration of 554 μM in the presence of 1:80 molar ratio of MMP-3 lacking the hemopexin-like domain (MMP-3ΔC) and 1 mM 4-aminophenylmercuric acetate for 90 min at 37 °C. The activation mixture was directly applied to a Sephadex S-200 gel filtration column (diameter 26 mm, length 900 mm) in 50 mM Tris–HCl (pH 7.5), 150 mM NaCl, 10 mM CaCl_2_, 0.02% (w/v) sodium azide, to separate the “active” form of MMP-1 (E200A) from MMP-3ΔC, 4-aminophenylmercuric acetate, and remnants of the pro-peptide. Fractions containing MMP-1 (E200A) were pooled and concentrated using a Vivacell 250 ml with a 5 kDa cutoff membrane, followed by a Vivaspin 20 with a 5 kDa membrane (Vivascience). The protein was stored at room temperature as incubation at 4 °C resulted in precipitation.

### Crystallisation, data collection and processing

MMP-1 (E200A) was crystallised using the hanging drop vapour-diffusion method. The protein (2 μl at a concentration of 21 mg/ml) was mixed with 2 μl of the reservoir solution containing 0.1 M Tris (pH 7.5), 1.5 M ammonium formate and 10% (w/v) polyethylene glycol (PEG) 8000. Crystals appeared and grew to their maximum size within two weeks at 16 °C. A cryoprotectant solution prepared by supplementing the reservoir with 25% (v/v) glycerol enabled the crystals to be flash-frozen in liquid nitrogen.

Flash-cooled MMP-1 (E200A) crystals were used to collect diffraction data to a resolution of 2.67 Å on PX 14.1 at the Synchrotron Radiation Source, Daresbury (UK). The data were processed and scaled using HKL2000.^25^ The crystals belong to the space group *P*3_2_21, with cell dimensions of *a* = *b* = 138.48 Å and *c* = 110.05 Å. There are two monomers in the asymmetric unit with solvent content of about 60%. Data reduction using the program TRUNCATE^26^ estimated an overall *B*-factor of 63.1 Å^2^/Da from the Wilson plot. Details of the data processing statistics are presented in Table 1.

### Structure determination

The solution for the MMP-1 (E200A) structure was found using the program PHASER.^26^ Human proMMP-1 (PDB code: 1SU3)^13^ was used as the search model with the pro-peptide domain removed from the structure. Log-likelihood gain (LLG) of 161 for the first solution increased to an LLG value of 1091 when the second monomer in the asymmetric unit was located indicating the solutions were indeed the right ones.

### Refinement

Crystallographic refinement was carried out using the program CNS^27^ at 2.67 Å resolution against 85.1% of the measured data. A test set of random reflections of 2.2% was excluded from the full data set for cross-validation purposes by calculating the free *R*-factor (*R*_free_) to monitor refinement trend.^28^ Initial round of refinement with the two monomers found by PHASER^26^ resulted in an *R*_cryst_ of 45.1% and an *R*_free_ of 50.6%. Once the six N-terminal residues in both the monomers were built in and all the ions (two zinc ions and four calcium ions per monomer) were added and the model subjected to simulated annealing, the *R*_cryst_ and the *R*_free_ dropped to 31% and 38%, respectively. Iterative cycles of refinement (energy minimisation, simulated annealing and individual temperature factor (*B*-factor) refinement) using CNS^27^ and model building with reference to 2*F*_o_-*F*_c_ and *F*_o_-*F*_c_ maps using the program Coot^29^ progressively improved the phases. In the final stages of refinement, water molecules with peaks greater than 3σ in the *F*_o_-*F*_c_ maps and those within hydrogen bonding distances from appropriate atoms were incorporated into the structure.

The final refined structure at 2.67 Å resolution has an *R*_cryst_ of 22.3% and an *R*_free_ of ∼26%. Both monomers, chains A and B have the full complement of the amino acids: from Phe81 to Cys447. Residues 117, 356, 369, 385, 386 and 424 of chain A and residues 257, 272, 287, 424 and 446 of chain B have been modelled as alanine residues because of the lack of visible electron density beyond Cβ atom. Analysis of the Ramachandran plot using the program PROCHECK^26^ indicated that ∼81% of the residues are in the most favourable region of the ϕ-φ plot and about 17% lie in the additional allowed region. The structure also consists of 198 water molecules. Details of the refinement statistics are given in Table 1.

### Protein Data Bank accession codes

The atomic coordinates and the structure factors of the human MMP-1 (E200A) have been deposited with the RCSB Protein Data Bank (accession codes 2CLT and R2CLTSF, respectively).
